# The Bioinformatics Analysis of Aldosterone-Producing Adenoma and Verification of Differentially Expressed Genes

**DOI:** 10.1155/2021/4926323

**Published:** 2021-10-12

**Authors:** Yinjie Gao, Xiaosen Ma, Huiping Wang, Yunying Cui, Yushi Zhang, Min Nie, Anli Tong

**Affiliations:** ^1^NHC Key Laboratory of Endocrinology (Peking Union Medical College Hospital), Department of Endocrinology, Peking Union Medical College Hospital, Peking Union Medical College, Chinese Academy of Medical Sciences, Beijing 100730, China; ^2^Department of Urology, Peking Union Medical College Hospital, Peking Union Medical College, Chinese Academy of Medical Sciences, Beijing 100730, China

## Abstract

**Purpose:**

Previous studies have investigated the transcriptional modulations of aldosterone overproduction of aldosterone-producing adenomas (APAs). We aimed to systematically study the genes and pathways associated with molecular mechanism underlying APA by bioinformatics analysis and experimental validation for the expression profile.

**Methods:**

This study was performed based on three gene expression profiles (GSE64957, GSE8514, and GSE60042). Differentially expressed gene (DEG) investigation, function and pathway enrichment analysis, and protein-protein interaction (PPI) network analysis were performed by the bioinformatics analysis. For the validation with quantitative PCR, tissues from 11 patients with nonfunctioning adrenal adenoma (NFA) and 13 with APA were included in our cohort.

**Results:**

In this study, the bioinformatics analysis was performed and 182 upregulated and 88 downregulated DEGs were identified. As expected, the upregulated DEGs were primarily involved in calcium ion homeostasis (*p* = 2.00X10^−4^). In the KEGG pathway analysis, calcium signaling pathway (*p* = 4.38X10^−6^) and the aldosterone synthesis and secretion (*p* = 8.73X10^−6^) were enriched. Moreover, quantitative PCR was performed to detect the expression of 7 upregulated genes (PCP4, ATP2A3, CYP11B2, CLCN5, HTR4, VDR, and AQP2) among the intersection of DEGs. The mRNA levels of CYP11B2, HTR4, and AQP2 were significantly increased in APA samples compared to NFA (24.420 folds of NFA, *p* < 0.001; 3.753 folds of NFA, *p* = 0.002; and 11.487 folds of NFA, *p* = 0.018).

**Conclusion:**

In summary, the present study showed several candidate genes with high expression from bioinformatics analysis and our cohort. Also, the DEGs were enriched in aldosterone synthesis and secretion and calcium signaling pathway as expected.

## 1. Introduction

Primary aldosteronism (PA) is the most common form of endocrine hypertension with a prevalence of 5–20% in patients with hypertension and is characterized by the excessive production of aldosterone [1, 2]. PA is mainly caused by either the aldosterone-producing adenoma (APA) or bilateral adrenal hyperplasia (BAH) [3].

Over the last decade, several studies investigated the gene expression profile of APAs compared to normal adrenals or adjacent adrenal cortexes with the aim of identifying transcriptional modulations of aldosterone overproduction [4, 5]. Genome-wide expression (microarray) and RNA-sequencing analysis (RNA-Seq) have become commonplace in the examination of gene expression of APA [6, 7]. Numerous genes, including the ones encoding steroidogenic enzymes such as CYP11B2, CYP11B1, CYP21A1, CYP11A1, CYP17A, and HSD3B2, few genes involved in calcium signaling or endoplasmic reticulum calcium storage such as CALM2, CALR, and CAMK-I, and several G-protein-coupled hormone receptors such as receptors of GnRH, LH, vasopressin, and serotonin, have been identified in previous studies as differentially expressed in APAs and the adrenal cortexes [5, 8].

**Figure 1:**
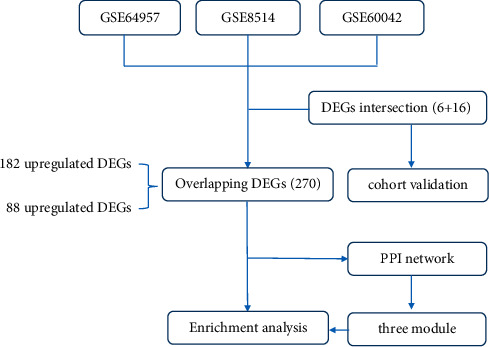
Workflow diagram.

In the present research, bioinformatics analysis and experimental validation for the expression profile of APAs compared with controls were studied. The workflow diagram is given in Figure 1. First, the bioinformatics analysis was performed based on several gene expression profiles. Differentially expressed gene (DEG) investigation, function and pathway enrichment analysis, and protein-protein interaction (PPI) network analysis were performed. We aimed to systematically investigate potential genes and pathways associated with the disease progression, which may aid in elucidating the molecular mechanism underlying APA. Additionally, several DEGs from databases were then verified in our cohort with 13 tissue samples from APA and 11 from nonfunctioning adrenal adenoma (NFA).

## 2. Materials and Methods

### 2.1. Data Resource

Gene expression profile data (accession no. GSE64957, GSE8514, and GSE60042) were downloaded from the Gene Expression Omnibus (GEO) database (http://www.ncbi.nlm.nih.gov/geo/). GSE64957 and GSE8514 datasets were produced on a GPL570 [HG-U133_Plus_2] Affymetrix Human Genome U133 Plus 2.0 Array platform, and GSE60042 was produced on a GPL14550 Agilent-028004 SurePrint G3 Human GE 8 × 60 K Microarray platform. A total of 47 tissue samples from APA patients and 39 normal tissue samples from their adjacent adrenal glands (AAG) were included in these datasets.

### 2.2. Data Preprocessing and Differential Expression Analysis

DEGs were obtained from GEO databases by a way of GEO2R analysis (http://www.ncbi.nlm.nih.gov/geo/geo2r/). The adj. *p* < 0.05 and |logFC| > 1.0 were set as DEGs cutoff criterion.

The intersection DEGs of these three datasets and any two of them were further considered more carefully, and all of the DEGs covered by these datasets were used for the enrichment analysis.

### 2.3. Gene Ontology and Pathway Enrichment Analysis of DEGs

The Database for Annotation, Visualization and Integrated Discovery (DAVID, http://david.abcc.ncifcrf.gov/) has facilitated the transition from data collection to biological analysis. The Gene Ontology (GO) and Kyoto Encyclopedia of Genes and Genomes (KEGG) pathway enrichment analyses were performed by the KEGG Orthology Based Annotation System (KOBAS) online tool (http://kobas.cbi.pku.edu.cn/anno_iden.php). *p* < 0.01 was set as the cutoff criterion.

### 2.4. Integration of Protein-Protein Interaction (PPI) Network and Modules Selection

To identify potential interactions between DEGs, a PPI network was constructed based on protein interactions between DEGs. The score of each node was determined by degree centrality, where an increased score indicates a more important location within the network. The Search Tool for the Retrieval of Interacting Genes (STRING, http://string.embl.de/) database was used to construct the PPI network for DEGs. The network view summarizes the network of predicted associations for a particular group of proteins. The network nodes are proteins, and edges represent the predicted functional associations. The cutoff criterion of confidence score was set as > 0.7. Subsequently, the results were visualized using Cytoscape software. It has been previously demonstrated that genes from the same module in a PPI network serve similar roles and are implicated in the same biological functions. The submodules were obtained to explore the DEGs with similar functions and which pathways they were related to. The submodule of the PPI network was further identified using the MCODE tool using the following parameters: degree cutoff = 2, node score cutoff = 0.2, *k*-core = 2, and max. depth = 100. The enrichment analysis of every module was further performed by KOBAS online.

### 2.5. Subjects and Tissues in Our Cohort

Tissues from 11 patients with NFA and 13 with APA were included. The clinical and pathological diagnoses were made according to established criteria [3, 9]. Patients with NFA had normotension and no signs or symptoms of hormone excess, had normal serum potassium (K+) levels, and displayed normal suppression of serum cortisol after low-dose dexamethasone treatment. We included only those patients where the discovery was incidental. All patients with APA had hypertension and hypokalemia and were diagnosed on the basis of an elevated plasma aldosterone concentration, suppressed plasma renin activity, and computerized axial tomography. All of the APA patients were detected with somatic KCNJ5 mutations by sequencing and positive staining of CYP11B2 by immunohistochemistry (IHC). The tumor tissue samples were obtained from unilateral adrenalectomy and snap frozen using liquid nitrogen and stored at −80°C until use. The study received ethical approval from the ethics committee of Peking Union Medical College Hospital. Written informed consent was obtained from all the patients.

### 2.6. RNA Extraction and Real-Time Quantitative PCR (RT-qPCR)

Total RNA was extracted using the Qiagen RNeasy Mini Kit (74104, Qiagen, Hilden, Germany) according to the manufacturer's protocol. The quality and quantity of total RNA were determined using an ND-2000 spectrophotometer (NanoDrop Technologies, Wilmington, DE, USA). First-strand cDNA was synthesized from 1 *µ*g of total RNA using PrimeScript RT (PK0446, Takara, Kusatsu, Japan) and oligo (dT) primers. For the target genes including PCP4, ATP2A3, CYP11B2, CLCN5, HTR4, VDR, and AQP2 identified by bioinformatics analysis above, a double-stranded DNA dye, SYBR-Green, was used with 10 *µ*l of SYBR-Green PCR master mix (PK0445, Takara, Kusatsu, Japan) and 100 nmol of each primer. PCR was performed using the ABI 7500 Fast Real-Time PCR System (Applied Biosystems) with a total volume of 20 *µ*l/reaction following the reaction parameters recommended by the manufacturer. All reactions were performed in triplicate. The primers used are given in Supplementary 1. RT-qPCR was conducted to analyze the mRNA expression of different genes in the tissues from different groups. Gene expression was analyzed by relative quantitation with the 2^^−ΔΔCt^ method using GAPDH as an internal control. The results are expressed as the target/internal standard concentration ratio of each sample.

### 2.7. Statistical Analysis

Normally and nonnormally distributed continuous variables were presented as mean ± SD and median (interquartile range), respectively. Independent *t* tests and nonparametric tests (the Mann–Whitney test) with IBM SPSS Statistics 22.0 software were used to assess differences between APA and NFA patients for the clinical characteristics. The fold change of qPCR data between APA and NFA samples was converted to logarithms, and the differences were analyzed by nonparametric tests. *p* value < 0.05 was considered to be significant.

## 3. Results

### 3.1. DEGs in APA Samples Compared with Control AAG Samples

**Figure 2:**
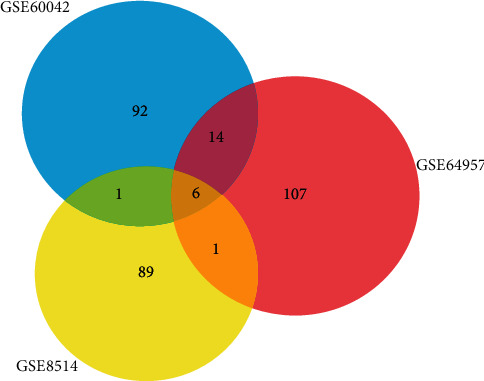
The Venn graph of three GEO databases.

As large amounts of data were included in the gene expression profiles, the original data were analyzed and filtered. A total of 182 upregulated and 88 downregulated DEGs were identified by GEO2R analysis. The intersection DEGs of the three datasets consisted of 6 genes, PCP4, ATP2A3, PPP4R4, CTNND2, CYP11B2, and CLRN1. More genes including IL17D, EDA2R, RAB3C, SCRN1, CLCN5, MTMR4, ABCB4, HTR4, QPCT, GBP2, NETO2, VDR, CBR1, ADAM23, FAM19A4, and AQP2 were covered in any two of these datasets. The Venn graph shows the intersection DEGs in Figure 2.

### 3.2. Functional Enrichment Analysis for DEGs

To further elucidate the roles of DEGs, GO functional and KEGG pathway enrichment analyses were performed. As expected, the upregulated DEGs were primarily involved in calcium ion homeostasis (GO: 0055074, *n* = 3, *p* = 2.00X10^−4^). Also, they also enriched in regulation of cardiac conduction (GO: 1903779), dendrite (GO: 0030425), synapse (GO: 0045202), and oligosaccharide metabolic process (GO: 0009311). In the KEGG pathway analysis, calcium signaling pathway (hsa04020, *n* = 8, *p* = 4.38X10^−6^, Supplementary 2 (I)) came first and the aldosterone synthesis and secretion (hsa04925, *n* = 6, *p* = 8.73X10^−6^, Supplementary 2 (I)) was the second one. The detailed information of upregulated DEG enrichment is shown in Table 1. As for downregulated DEGs, the most common enrichments were protein binding (GO: 0005515), extracellular space (GO: 0005615), collagen-containing extracellular matrix (GO: 0062023), extracellular region (GO: 0005576), and extracellular exosome (GO: 0070062). Also, the downregulated DEGs were enriched in the KEGG pathways of cancer (hsa05200) and proteoglycans in cancer (hsa05205). The detailed information of downregulated DEG enrichment is shown in Table 2.

### 3.3. PPI Network Construction from DEGs

**Figure 3:**
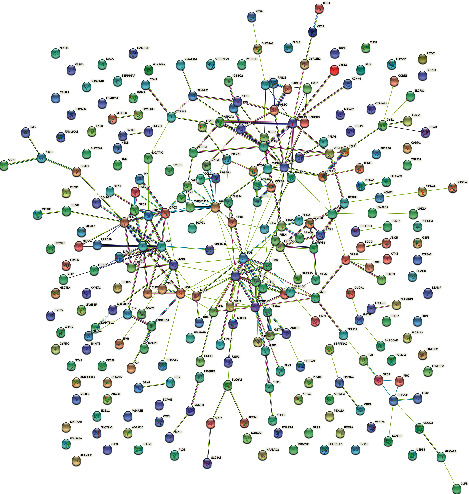
The protein-protein interaction network with all of the 270 differentially expressed genes. The proteins were represented by the nodes, and the predicted functional associations were represented by the edges.

The DEGs were further analyzed using the STRING database to construct the PPI network, and the general PPI network with all of the 270 DEGs is shown in Figure 3. There were 182 nodes and 285 edges with an average node degree of 3.13 in the constructed network. Furthermore, the constructed PPI network was exported into Cytoscape software and subjected to the submodule PPI network construction using the MCODE tool. As shown in Supplementary 3 ((Ia) for module 1, (Ib) for module 2, and (Ic) for module 3), three submodules with MCODE score greater than 4.0 were identified from the constructed PPI network. There were 5 nodes and 9 interactions in module 1, 15 nodes and 31 interactions in module 2, and 9 nodes and 14 interactions in module 3. In module 3, the DEGs were primarily involved in positive regulation of cytosolic calcium ion concentration (GO: 0007204, *n* = 3, *p* = 1.45X10^−6^), including PTGFR, CCKBR, and TACR1 genes. In the KEGG pathway analysis of module 3, calcium signaling pathway (hsa04020, *n* = 4, *p* = 7.60X10^−8^) with PTGFR, CCKBR, HTR2B, and TACR1 genes was enriched firstly. The detailed information of module 3 enrichment analysis is shown in Supplementary 3 (II). Other modules (modules 1 and 2) were not enriched successfully due to the few nodes.

### 3.4. Clinical Manifestations of Study Participants

Thirteen patients with diagnosed APA (age: 27–69 years) and 11 patients with NFA (as control group) were recruited in this study, which are used to confirm the mRNA expression of several DEGs by RT-qPCR. Clinical characteristics of these patients are summarized in Supplementary 4. The systolic blood pressure (SBP), diastolic blood pressure (DBP), and aldosterone of APA group were all higher, and the plasma K+, PRA, and tumor size were lower than that in NFA group. The above results showed that the clustering on clinical manifestations of these two groups was obvious, which is suitable for the subsequent RT-qPCR analysis.

### 3.5. The mRNA Expression of Several DEGs by RT-qPCR

**Figure 4:**
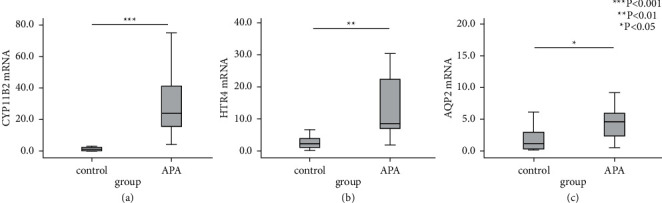
The mRNA expression levels of 3 upregulated genes CYP11B2, HTR4, and AQP2 between APAs and NFAs. *Y* axis represents the test group (APA) and control group (NFA); *X* axis represents the relative expression (fold change) of each mRNA. ^*∗∗∗*^, *p* < 0.001, ^*∗∗*^, *p* < 0.01, and ^*∗*^, *p* < 0.05. The lines from the top to bottom show the upper margin, the upper quartile *Q*3, the median, the lower quartile *Q*1, and the lower margin separately. (a) The mRNA expression of CYP11B2, (b) the mRNA expression of HTR4, and (c) the mRNA expression of AQP2.

To confirm the results of bioinformatics analysis, RT-qPCR was performed to detect the mRNA expression of 7 upregulated genes (PCP4, ATP2A3, CYP11B2, CLCN5, HTR4, VDR, and AQP2) among the intersection of DEGs, which related to aldosterone synthesis and secretion and calcium signaling regulation (the proteins encoded by the above seven genes and their biological functions are shown in Supplementary 5). The mRNA levels of CYP11B2, a well-known upregulated gene, were also tested positively in our cohort (24.420 folds of NFA,*p* < 0.001). And also, the HTR4 and AQP2 were significantly increased in APA samples compared to NFA (3.753 folds of NFA, *p* = 0.002, and 11.487 folds of NFA, *p* = 0.018). The fold changes, *p* values of all the 7 genes, and the box plots of 3 upregulated genes by RT-qPCR are shown in Table 3 and Figure 4.

## 4. Discussion

In this study, the bioinformatics analysis was performed and 182 upregulated and 88 downregulated DEGs were identified. As expected, the upregulated DEGs were enriched in the calcium signaling pathway and the aldosterone synthesis and secretion. The intersection DEGs of different GEO databases, which are also related to the pathways above, included PCP4, ATP2A3, CYP11B2, CLCN5, HTR4, VDR, and AQP2. Furthermore, the mRNA levels of CYP11B2, HTR4, and AQP2 were significantly increased in 13 APA samples compared to 11 NFA samples from our cohort, which confirmed the high expression of these genes and the important role in the occurrence and development in APA.

This is the first study to explore the potential mechanism of APA through the method of bioinformatics analysis. With the comprehensive consideration of many research studies about expression profiles in APA, the reliability of the final results was increased. By the functional enrichment analysis of DEGs, we further confirmed the relationship between APA and regulated pathways, including aldosterone synthesis and secretion, calcium signaling pathway, G-protein-coupled receptor signaling pathway, cAMP signaling pathway, positive regulation of cytosolic calcium ion concentration, and regulation of cardiac conduction, which were consistent with the previous studies of APA transcriptome profiles [7, 10, 11]. Moreover, several upregulated pathways, such as oligosaccharide metabolic process, response to muscle stretch, neurogenesis (dendrite or synapse), neuroactive ligand-receptor interaction, positive regulation of transcription (DNA-templated), and DNA-binding transcription activator activity (RNA polymerase II-specific) were enriched in APA samples, which were discovered for the first time. As for the downregulated DEGs, we found that the pathways of cancer were enriched in these datasets. It might be one of the reasons why the APA has the slower speed of growth and proliferation and smaller tumor size than NFA.

There were 22 genes which were highly expressed from at least two GEO databases about APA by our bioinformatics analysis. The most prominent of these was CYP11B2 gene, which encodes aldosterone synthase and catalyzes the multistep reaction of deoxycorticosterone to produce aldosterone. CYP11B2 is considered as a marker of aldosterone synthesis and widely believed with upregulated expression in APA. Besides, the other three DEGs, PCP4, HTR4, and VDR, have also been reported to be involved in the occurrence and development of APA [12–14], and among them, the upregulated expression of HTR4 was verified by the qPCR in this study. HTR4 is a serotonin receptor subtype known to be expressed in adrenal gland, and serotonin acted on the receptor can increase aldosterone secretion in vivo and in vitro [15–17]. In addition, several other DEGs that are related to calcium signaling pathway and aldosterone secretion have not been reported to participate in the progression of APAs yet, such as ATP2A3, CLCN5, and AQP2. In this study, we found them highly expressed in APA firstly. Also, only the last one, AQP2, was verified in our cohort. The AQP2 is an ADH sensitive aquaporin. The recent studies said that the binding of ADH and receptor can cause a transient increase of intracellular calcium [18, 19], which followed the increased aldosterone secretion. This phenomenon of upregulated expression of AQP2 in APAs was also found by other researchers without detailed elucidation [8, 20]. However, Niu et al. found that AQP2 was expressed in all the adrenal medullary tumors, but not in adrenal cortical tumors, which reflected the maintenance of water metabolism via AQP2 during tumorigenesis [21]. Therefore, whether the gene is related to the occurrence of APAs remains to be further studied.

Perhaps due to the limitation of samples and the specificity of selected tissues, the high expression of the other genes was not verified in our cohort and further research studies are needed to confirm the gene expression from the related pathways. In this study, we made NFA tissues as the control group of APAs, which were used more for discussing the secretion function and the related mRNA expression. Nevertheless, AAG tissues were used widely as the normal control in the previous studies and GEO datasets. Although reducing the difference of the intragroup to a certain extent, it was not easy to obtain the AAG samples from single zona glomerulosa of adrenal gland accurately. The cells in distinct zones might have different steroid hormone expression profiles.

Several limitations to our study should be acknowledged. First of all, the microarray data we included were generated from the NCBI website [16, 22, 23]. Besides the qPCR verification for mRNA expression of several significant genes, other experiments are needed to confirm their impacts on APA. Second, the three GEO databases all used AAG as control to explore the differences of transcription. In this study, we recruited the patients with NFA as control. It might be one of the reasons why the expression of some genes did not show upregulation as we found in the databases.

## 5. Conclusions

In summary, the present study showed candidate genes with high expression, and part of them was verified in our cohort, which might be involved in the occurrence and development of APA. Also, all the DEGs were enriched in aldosterone synthesis and secretion and calcium signaling pathway. Nevertheless, future studies are warranted to determine the detailed molecular mechanisms underlying APA more than bioinformatics analysis.

## Figures and Tables

**Table 1:** The detailed information of upregulated DEG enrichment in the functional enrichment analysis of APAs.

ID	Analysis type	Process	Count	*p* value	Genes
hsa04020	KEGG pathway	Calcium signaling pathway	8	4.38X10^−6^	CCKBR, PHKA1, HTR4, HTR2B, TACR1, ATP2A3, ATP2B2, ATP2B3
hsa04925	KEGG pathway	Aldosterone synthesis and secretion	6	8.73X10^−6^	MC2R, CYP11B2, ATP2B3, PDE2A, ATP2B2, ATP1B1
hsa04024	KEGG pathway	cAMP signaling pathway	7	7.52X10^−5^	CNGB3, MC2R, HTR4, ATP2B3, ATP2A3, ATP2B2, ATP1B1
GO: 1903779	Gene ontology	Regulation of cardiac conduction	4	1.37X10^−4^	ATP1B1, ATP2A3, ATP2B3, ATP2B2
GO: 0030425	Gene ontology	Dendrite	9	1.71X10^−4^	RELN, KCND3, HTR4, CYP46A1, SLC4A8, HTR2B, CTNND2
GO: 0045202	Gene ontology	Synapse	9	1.71X10^−4^	GPC4, CHRNA5, CYFIP2, PMP22, HTR4, GJC1, HTR2B, CADM1, EEF1A2
GO: 0009311	Gene ontology	Oligosaccharide metabolic process	3	1.77X10^−4^	ST3GAL6, NAGA, ST8SIA5
GO: 0055074	Gene ontology	Calcium ion homeostasis	3	2.00X10^−4^	SGCD, WFS1, SNX10

DEG, differentially expressed gene; APA, aldosterone-producing adenoma.

**Table 2:** The detailed information of downregulated DEG enrichment in the functional enrichment analysis of APAs.

ID	Analysis type	Process	Count	*p* value	Genes
hsa05200	KEGG pathway	Pathways in cancer	9	3.49X10^−6^	MMP2, PRKCA, GSTA2, EPAS1, PTGER3, FOS, JUN, GSTA5, HGF
hsa05205	KEGG pathway	Proteoglycans in cancer	6	7.90X10^−6^	LUM, MMP2, DCN, PRKCA, GPC3, HGF
hsa05166	KEGG pathway	Human T-cell leukemia virus 1 infection	6	1.20X10^−5^	HLA-DMA, ZFP36, FOS, JUN, ETS2, EGR1
GO: 0005515	Gene ontology	Protein binding	62	7.04X10^−15^	ZNHIT2, USP2, NAALAD2…
GO: 0005615	Gene ontology	Extracellular space	23	5.70X10^−13^	MMP2, SEMA3C, HBB…
GO: 0062023	Gene ontology	Collagen-containing extracellular matrix	13	3.59X10^−12^	NID1, BGN, DCN…
GO: 0005576	Gene ontology	Extracellular region	21	6.49X10^−10^	LUM, BGN, DCN…
GO: 0070062	Gene ontology	Extracellular exosome	21	5.66X10^−9^	LUM, BGN, PRKCA…

DEG, differentially expressed gene; APA, aldosterone-producing adenoma.

**Table 3:** The mRNA expression in samples of APA compared to ones of NFA.

	Gene name	Fold change	*p* value
1	PCP4	1.593	0.331
2	ATP2A3	1.587	0.252
3	CYP11B2	24.420	<0.001
4	CLCN5	0.326	0.228
5	HTR4	3.753	0.002
6	VDR	1.754	0.424
7	AQP2	11.487	0.018

## Data Availability

All the data and materials used have been contained in the public database and the tables, figures, and supplementary materials of this article.

## Supplementary Materials

Supplementary Materials
*Supplementary 1*. The primers used in real-time RT-PCR. *Supplementary 2*. The pathways' diagrams of KEGG analysis. *Supplementary 3*. The submodule of protein-protein interaction network and the enrichment analysis of module 3. *Supplementary 4*. Clinical characteristics of 11 patients with NFA and 13 patients with APA. *Supplementary 5*. The proteins encoded by seven genes from DEGs and their biological functions.
