# Supplementary material for: The Bioinformatics Analysis of Aldosterone-Producing Adenoma and Verification of Differentially Expressed Genes
Source: Int J Endocrinol. 2021 Oct 12;2021:4926323. doi: 10.1155/2021/4926323 (PMC8526198; doi:10.1155/2021/4926323)

Supplementary Material 2. The pathways’ diagrams of KEGG analysis

(a) The calcium signaling pathway in the KEGG pathway analysis


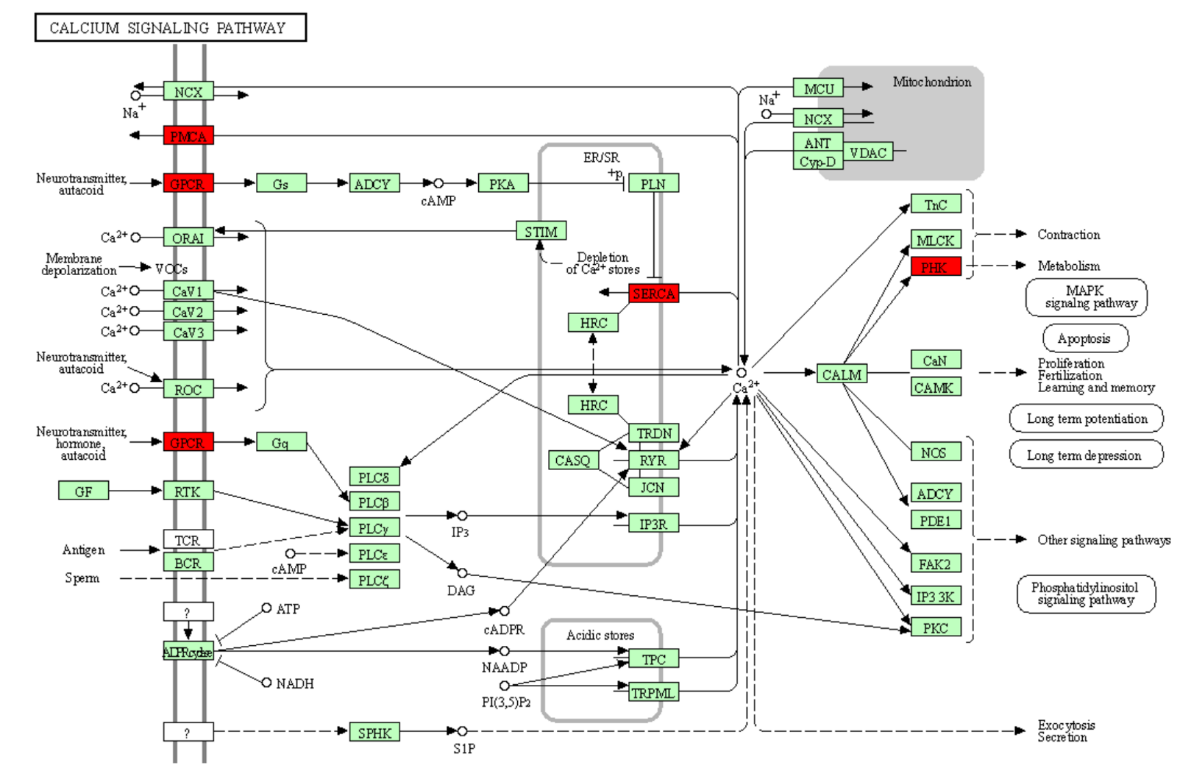


(b) The aldosterone synthesis and secretion in the KEGG pathway analysis


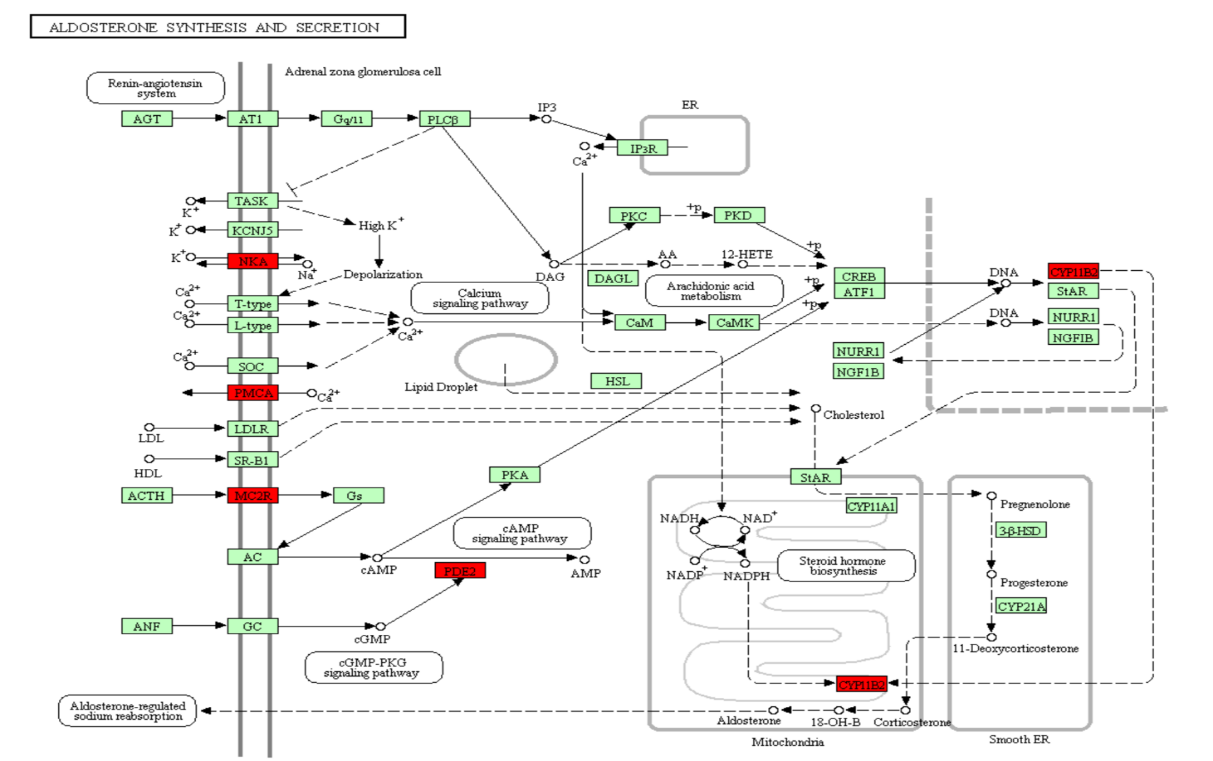

Supplement: Supplementary Materials — Supplementary 1. The primers used in real-time RT-PCR. Supplementary 2. The pathways' diagrams of KEGG analysis. Supplementary 3. The submodule of protein-protein interaction network and the enrichment analysis of module 3. Supplementary 4. Clinical characteristics of 11 patients with NFA and 13 patients with APA. Supplementary 5. The proteins encoded by seven genes from DEGs and their biological functions. [file 4926323.f1.zip › 4926323.f1/Supplementary Material 2 (1).docx]
