# Supplementary material for: The Bioinformatics Analysis of Aldosterone-Producing Adenoma and Verification of Differentially Expressed Genes
Source: Int J Endocrinol. 2021 Oct 12;2021:4926323. doi: 10.1155/2021/4926323 (PMC8526198; doi:10.1155/2021/4926323)
Supplement: Supplementary Materials — Supplementary 1. The primers used in real-time RT-PCR. Supplementary 2. The pathways' diagrams of KEGG analysis. Supplementary 3. The submodule of protein-protein interaction network and the enrichment analysis of module 3. Supplementary 4. Clinical characteristics of 11 patients with NFA and 13 patients with APA. Supplementary 5. The proteins encoded by seven genes from DEGs and their biological functions. [file 4926323.f1.zip › 4926323.f1/Supplementary Material 3 (1).docx]

Supplementary Material 3. The sub-module of protein-protein interaction network and the enrichment analysis of Module 3

(a) The sub-module from protein-protein interaction network


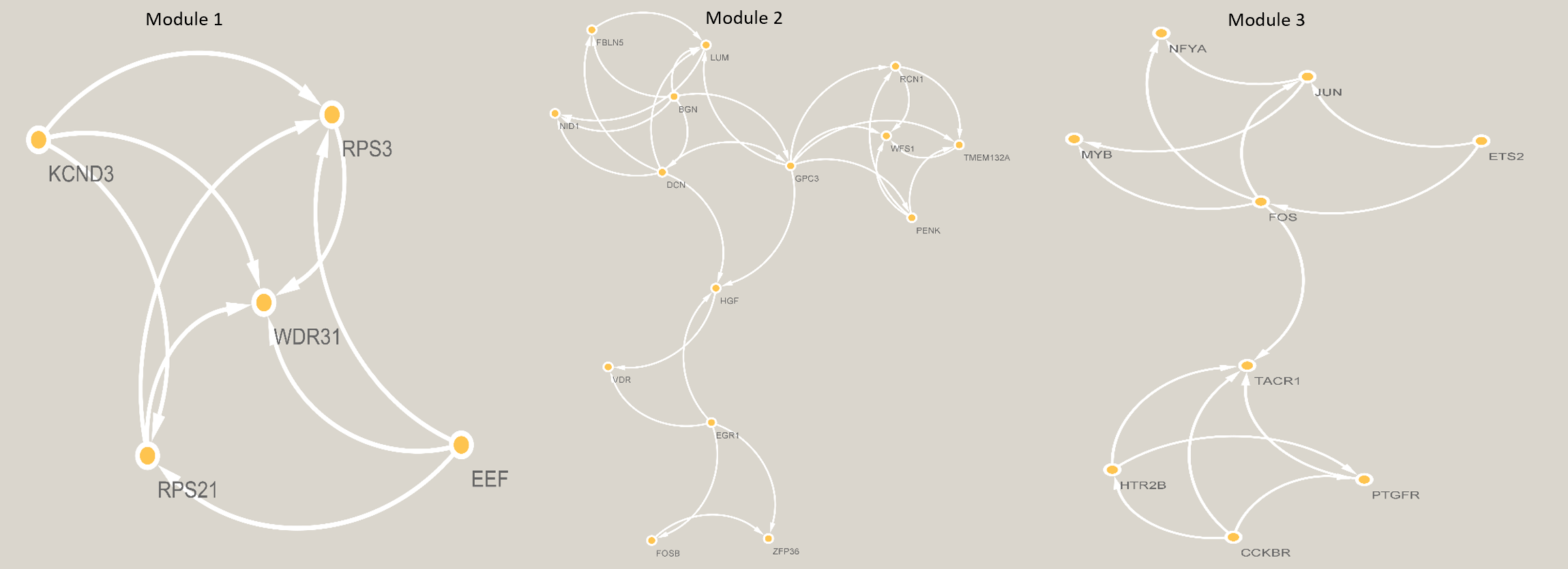


(b) The detail information of Module 3 enrichment analysis of APA

| ID | Analysis type | Process | Count | P-value | Genes |
| --- | --- | --- | --- | --- | --- |
| hsa04020 | KEGG Pathway | Calcium signaling pathway | 4 | 7.60X10-8 | PTGFR, CCKBR, HTR2B, TACR1 |
| hsa04080 | KEGG Pathway | Neuroactive ligand-receptor interaction | 4 | 6.89X10-7 | PTGFR, CCKBR, HTR2B, TACR1 |
| hsa05166 | KEGG Pathway | Human T-cell leukemia virus 1 infection | 3 | 1.46X10-5 | FOS, ETS2, JUN |
| GO: 0045893 | Gene Ontology | positive regulation of transcription, DNA-templated | 5 | 7.04X10-8 | NFYA, FOS, MYB, ETS2, JUN |
| GO: 0007200 | Gene Ontology | phospholipase C-activating G protein-coupled receptor signaling pathway | 3 | 3.29X10-7 | HTR2B, CCKBR, TACR1 |
| GO: 0001228 | Gene Ontology | DNA-binding transcription activator activity, RNA polymerase II-specific | 4 | 2.44X10-6 | NFYA, FOS, MYB, JUN |
| GO: 0007204 | Gene Ontology | positive regulation of cytosolic calcium ion concentration | 3 | 1.45X10-6 | PTGFR, CCKBR, TACR1 |
| GO: 0035994 | Gene Ontology | response to muscle stretch | 2 | 6.34X10-6 | FOS, JUN |

APA: aldosterone-producing adenoma
