# Supplementary material for: The Bioinformatics Analysis of Aldosterone-Producing Adenoma and Verification of Differentially Expressed Genes
Source: Int J Endocrinol. 2021 Oct 12;2021:4926323. doi: 10.1155/2021/4926323 (PMC8526198; doi:10.1155/2021/4926323)
Supplement: Supplementary Materials — Supplementary 1. The primers used in real-time RT-PCR. Supplementary 2. The pathways' diagrams of KEGG analysis. Supplementary 3. The submodule of protein-protein interaction network and the enrichment analysis of module 3. Supplementary 4. Clinical characteristics of 11 patients with NFA and 13 patients with APA. Supplementary 5. The proteins encoded by seven genes from DEGs and their biological functions. [file 4926323.f1.zip › 4926323.f1/Supplementary Material 5 (1).docx]

Supplement Material 5. The proteins encoded by seven genes from DEGs and their biological functions

| Gene | Protein | Biological functions |
| --- | --- | --- |
| PCP4 | Purkinje cell protein 4 | Calcium ion binding, calmodulin binding, protein binding |
| ATP2A3 | ATPase sarcoplasmic/endoplasmic reticulum Ca2+ transporting 3 | ATP binding, ATPase activity, calcium ion transmembrane transporter activity, calcium-dependent ATPase activity |
| CYP11B2 | cytochrome P450 family 11 subfamily B member 2 | Corticosterone 18-monooxygenase activity, heme binding, iron ion binding, steroid 11-beta-monooxygenase activity, steroid hydroxylase activity |
| CLCN5 | chloride voltage-gated channel 5 | ATP binding, chloride channel activity (when the chloride current of CLC-5 channel raise cause of the upregulated expression of CLCN5, the calcium reabsorption regulated by PTH and calcitonin might increase, which lead to intracellular high calcium), identical protein binding, protein binding, solute: proton antiporter activity, voltage-gated chloride channel activity |
| HTR4 | 5-hydroxytryptamine receptor 4 | G protein-coupled serotonin receptor activity (serotonin acts on HTR4 could increase aldosterone secretion), neurotransmitter receptor activity, protein binding, serotonin binding |
| VDR | vitamin D receptor | DNA binding, calcitriol binding, lithocholic acid binding, nuclear receptor activity, retinoid X receptor binding, contributes to vitamin D response element binding (a regulator of extracellular Ca2+, could regulate steroid production including aldosterone), zinc ion binding |
| AQP2 | aquaporin 2 | Glycerol transmembrane transporter activity, protein binding, water channel activity (calcium sensitive water channel), water transmembrane transporter activity |
